# Mucinous Ovarian Tumors With Anaplastic Mural Nodules: Case Report

**DOI:** 10.3389/fmed.2021.753904

**Published:** 2021-12-14

**Authors:** Xinxin Zou, Hao Huang, Qingyu Zhang, Zhen Ma, Yumei Chen, Weifeng Wu, Aizhen Fu

**Affiliations:** ^1^Department of Obstetrics and Gynecology, Affiliated Hospital of Guangdong Medical University, Zhanjiang, China; ^2^Graduate School of Guangdong Medical University, Zhanjiang, China; ^3^The Marine Biomedical Research Institute, Guangdong Medical University, Zhanjiang, China

**Keywords:** ovarian cancer, mucinous ovarian tumors, mural nodules, anaplasia, carcinoma

## Abstract

Ovarian mucinous cystic tumors may be associated with various types of mural nodules, which can be classified as benign or malignant (anaplastic carcinoma, sarcoma, carcinosarcoma). However, anaplastic malignant nodules have rarely been reported. Here, we present a case of a 35-year-old woman who presented with abdominal discomfort. Ultrasonography showed a large cystic mass in the pelvic and abdominal cavities measuring 337 × 242 mm. Abdominal computed tomography revealed upper anterior and posterior uterine pelvic cystic lesions based on multiple nodule partition walls and classes. During hospitalization, the patient underwent exploratory laparotomy, which revealed a poorly differentiated ovarian malignant tumor, and subsequent surgical excision was performed. The pathological analysis of the surgical samples of the right ovary revealed a mucinous ovarian tumor, while the mural nodules were classified as anaplastic carcinoma. After surgery, the patient started receiving chemotherapy. Unfortunately, the patient died 6 months later. Mucinous tumor occurring with an anaplastic carcinoma is rare, and the current diagnostic methods are not sufficient in providing an early and accurate diagnosis. Most patients are already in the advanced stage upon diagnosis and combined with poorly differentiated pathological features, the prognosis is extremely poor. Clinicians need to improve the clinical evaluation before surgery and conduct preoperative preparation and communication to improve the prognosis of patients as much as possible.

## Introduction

According to cancer statistics in 2018, epithelial ovarian cancer (EOC) is the main cause of morbidity and death in gynecological malignancies (1). Although treatment of gynecological malignancies has progressed with the use of surgery and chemotherapy, the 5-year survival rate remains at 25%, which is still very low (2). A mucinous cystic tumor with malignant mural nodules is rare. Zhang *et al*. have reported (3) that in the past 40 years, there have only been 75 cases of mucinous cystic tumors with anaplastic carcinoma, which is the most common malignant nodule, while 9 cases had sarcomatous mural nodules. Compared with high-grade EOC, the disease does not have accurate molecular markers and an established diagnostic criterion, which makes early diagnosis difficult. In addition, the disease rapidly progresses. Once diagnosed, the prognosis of the patient remains extremely poor despite surgery and chemotherapy. In this study, we presented a case, hoping for this case according to the diagnostic process and the lack of experience, we are in for the next time, we can make a more effective diagnosis and treatment.

## Case Presentation

**Figure 1:**
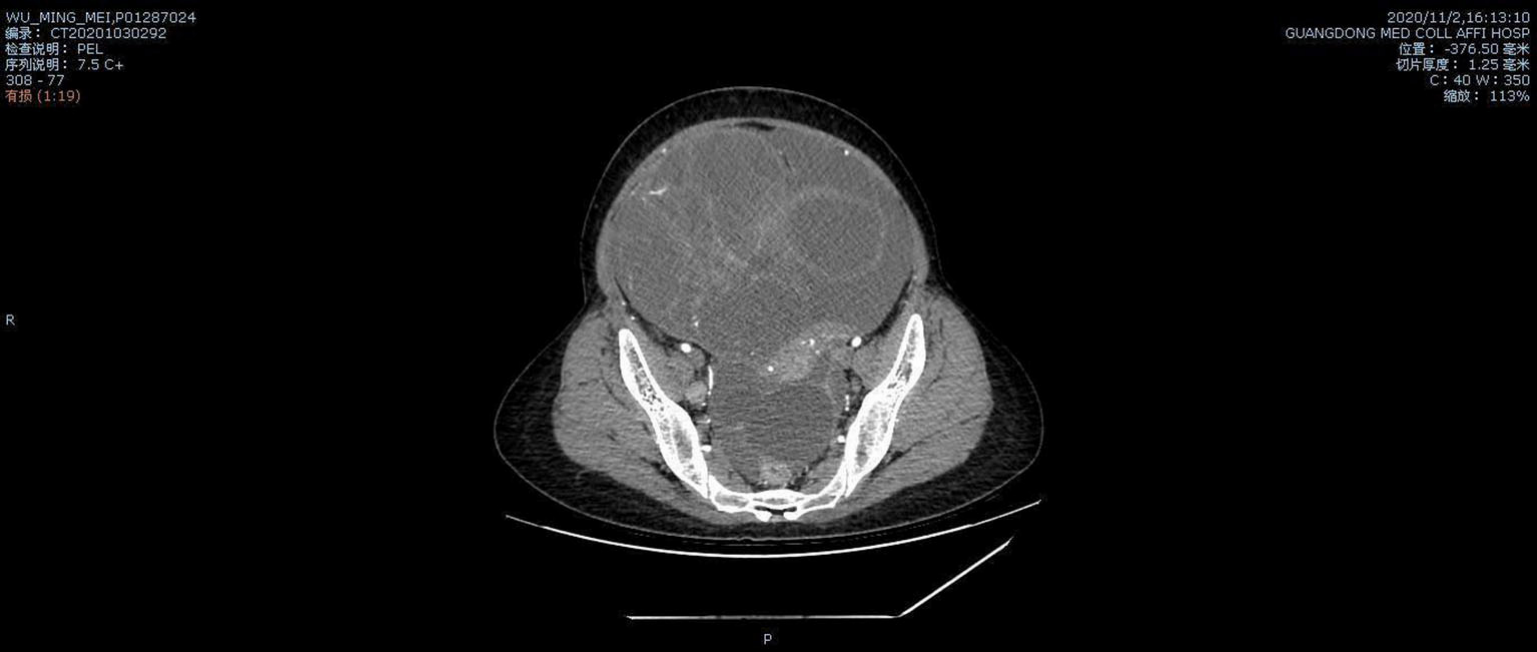
Pelvic CT result.

**Figure 2:**
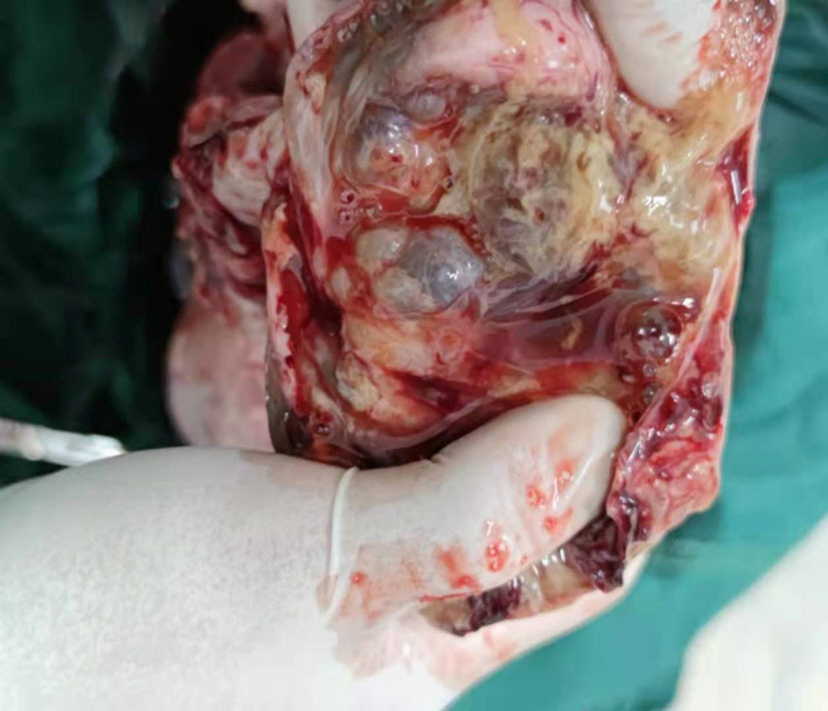
Macroscopic appearance of tumor.

**Figure 3:**
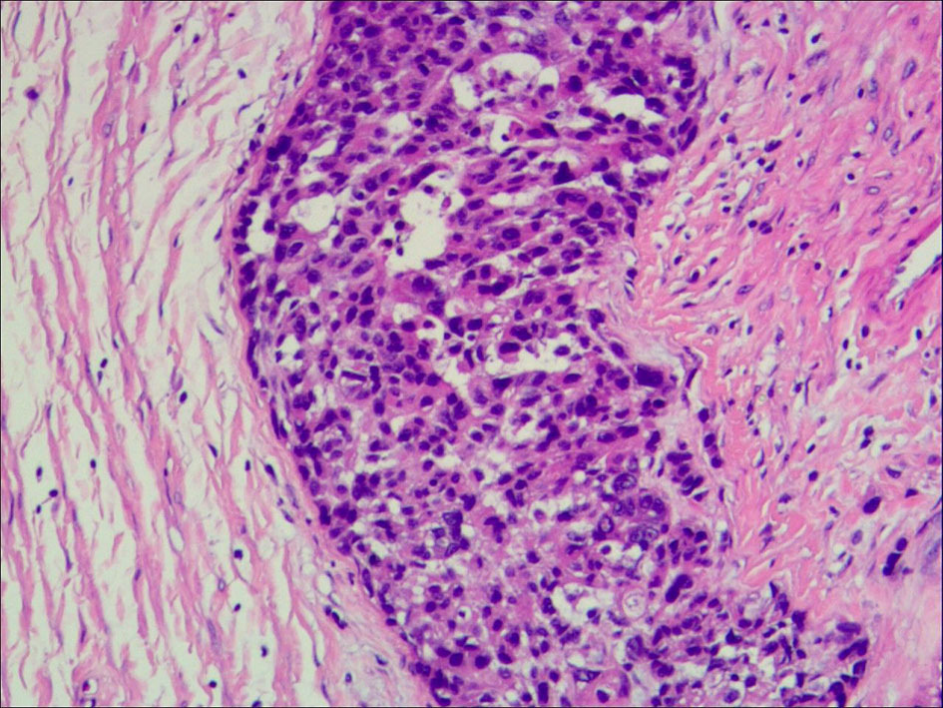
The histopathology results.

**Figure 4:**
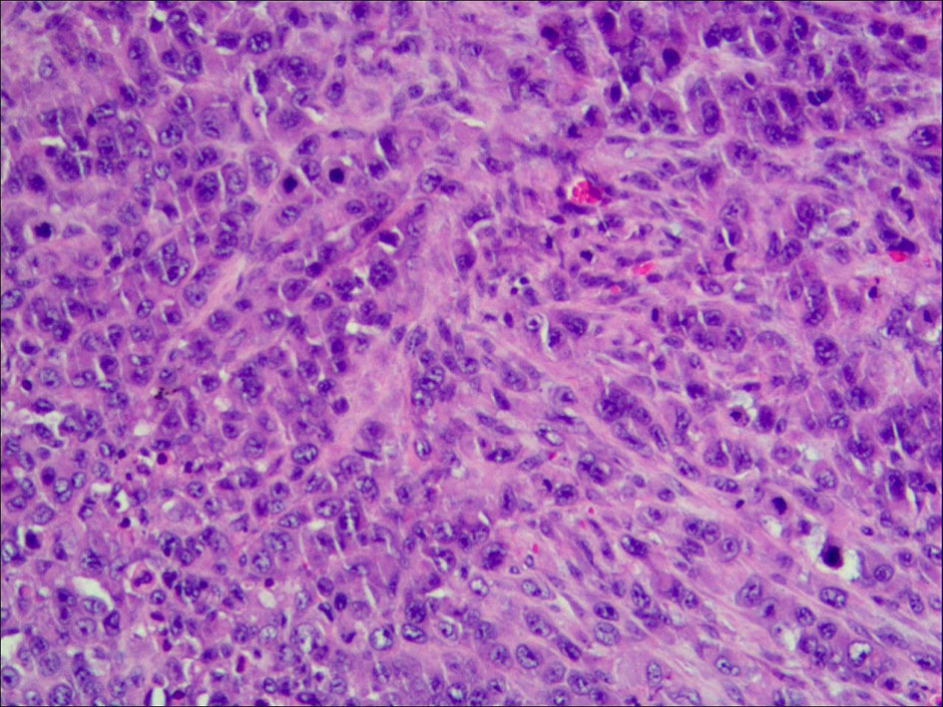
The tumor cells are arranged in broad.

A 35-year-old G0P0 presented with abdominal discomfort of 6 months duration. The patient has not been given special treatment in the past. Physical examination of the abdomen revealed mild tenderness and a hard palpable mass. The preoperative levels of serum cancer antigen 125 (CA-125), CA19-9, and human epididymal protein-4 (HE-4) were 322.9 U/ml, 766 U/ml, and 112.9 pmol/L, respectively. B-Ultrasonography (B-US) showed a huge mixed mass in the pelvic cavity measuring 337 × 242 mm. The mass had clearly defined boundaries, with most of the liquid-filled dark areas separated by strong light bands. Color Doppler flow imaging revealed a blood flow signal above the divider. The diagnosis based on the B-US was ovarian cystadenoma. Abdominal computed tomography results are as follows: a huge cystic space-occupying lesion can be seen in the anterior and upper part of the pelvic uterus. The lesions on the scan level are not complete. The scan range is about 284 × 205 mm on the larger level, the upper and lower diameter is about 151 mm (incomplete). The lesion shows multilocular cystic changes, the cyst wall is thin, multiple partitions can be seen, and several mural nodules can be seen, and the larger mural node is 28 × 23 mm, slight enhancement was seen on the enhanced scan. It seems that the blood supply artery came from the branch of the uterine and ovarian arteries. Large cystic space-occupying lesions with a size of about 89 mm were also seen behind the uterus. The wall is thin, and a sheet-like enhancement shadow can be seen at the left front. Bilateral ovaries showed unclearly. The anterior position of the uterus is obviously compressed, and the anterior and posterior cystic space-occupying parts seem not to be connected. The focus behind the uterus seems to be closely related to the left ovary. A large amount of effusion shadow can be seen in the pelvic cavity. Large lymph nodes with a size of about 17 mm can be seen next to the iliac vessels on the right side of the pelvic cavity × 12mm, enhanced scanning was more obvious. No abnormality is found in the pelvic bone structure (Figure 1). Exploratory laparotomy was performed, which revealed a pelvic mass measuring 33 × 20 cm with a smooth capsule that was filled with ~12,000 ml of fluid. The solid area was soft, grayish, and measured ~20 × 14 cm, while the cystic area measured ~10 × 8 cm and contained a jelly-like material. The inner wall of the sac was covered with white moss, and the wall thickness ranged from 0.1 to 0.6 cm (Figure 2). The omentum was immobile and had miliary nodules on its surface. Right adnexectomy, omentectomy, appendectomy, and pelvic lymph node dissection were concomitantly performed. The postoperative histopathology results showed the following: (1) Consider the mucinous tumor of the right ovary with mural nodules (anaplastic carcinoma) in combination with the results of immunohistochemistry; (2) see tumors in the momentum and peritoneum tissue; (3) see cancer metastasis in the right obturator foramen, right deep common iliac lymph nodes, right common iliac and right inguinal lymph nodes, and no metastasis. The immunohistochemistry results were as follows: Alk (D5F3)(–), CA125(–), CD30(–), CD38(–), CD99(–), CEA(–), CgA(–), CK(+), CK20(–), CK7(–), CR(partly +), estrogen receptor (ER)(–), GFAP(–), LCA(–), Melan– A(–), PAX8(–), progesterone receptor (PR)(–), S-100(–), Syn(+), vimentin(+), WT-1(–), and alpha-statin(–) (Figures 3, 4). The final diagnosis was a mucinous ovarian tumor with mural nodules and anaplastic carcinoma, FIGO (2014) Stage IIIC. After the operation, the patient underwent three courses of carboplatin and docetaxel intraperitoneal hyperthermic perfusion chemotherapy. Unfortunately, 6 months later, the patient died.

## Discussion

Despite being highly malignant ovarian tumors, there is a lack of effective diagnostic tools and molecular markers to confirm the diagnosis of mucinous ovarian tumors. Mucinous tumors usually occur in young women (4), and in recent years, studies on the biology of mucinous ovarian tumor molecules have increased. For mucinous ovarian tumors, the prognosis of anaplastic and sarcomatous tumors varies; therefore, dural nodules must be distinguished as anaplastic or sarcomatous tumors. Compared with other ovarian cancer tissue types, anaplastic tumors are usually immunohistochemically positive for vimentin and CAM5.2 (5); aggressive mEOCs have benign or atypical epithelial cell foci with the same tumor KRAS mutations (6). KRAS may be related to the degree of malignancy. However, sarcomatous tumors may produce the same immunohistochemistry results. AE1/AE3 can be used to distinguish these subtypes because it is only positive in anaplastic tumors (7). In our case, the preoperative levels of CA125 and HE-4 were significantly increased, in which the possibility of a higher malignancy needed to be considered before the operation. Further evaluation using electronic gastrointestinal endoscopy could have been performed, but the patient refused. In the meantime, the patients were so young that we ignored the possibility of malignant tumors. In this case, the preoperative evaluation and doctor-patient communication should have been improved. Surgery was performed based on the results of rapid freezing, which has limitations and passivity. According to the diagnosis and treatment guidelines, a comprehensive staged radical operation is required, the patient was unmarried and childless, hence, the left ovary and uterus were retained. The tumor cytoreductive surgery was performed. In the diagnosis and treatment of ovarian tumors in young women, the accuracy of the diagnosis determines the prognosis of the patient, and precautions must be taken before surgery. On the other hand, methods and techniques to accurately diagnose the disease are necessary. Regarding the immunohistochemistry results of the tumor being ER(–), PR(–), and PAX8(–), these are similar to a case reported by Mhawech-Fauceglia (8). However, the pathology department of our hospital did not perform immunohistochemical analysis of AE1 and AE3, making our diagnosis slightly weaker than that of Paulette's. On the other hand, CK(+), CR(partly +), Syn(+), and vimentin(+) can become molecular markers of the disease. It takes a long time to go. The patient underwent three courses of carboplatin and docetaxel intraperitoneal hyperthermic perfusion chemotherapy. However, she was refractory to treatment. From this case, we can see that the early age of onset of the disease, rapid progression, and poor prognosis. Therefore, a set of standard diagnosis and treatment principles is urgently needed to determine the diagnosis that should be made, how the patient's reproductive function can be ensured, and whether the patient's life should be prolonged, and to what extent.

Ovarian mucinous tumors represent a group of rare neoplasms with a still undefined cell of origin but with an apparent progression from benign to borderline to carcinoma (16). Ovarian cancers of mucinous and non-mucinous histology are significantly different with respect to clinical characteristics, survival and molecular alterations (17). Here, we summarized previously reported data on the disease, in which Table 1 shows us the poor prognosis of this disease. Early detection of these tumors results in a better prognosis in the later stages, whereas those detected later during the disease course carry a very poor prognosis. This case elucidates the manifestations and treatment of this type of ovarian cancer and summarized the experience of managing the disease based on the difficulties encountered during the diagnosis and treatment processes. Avoiding misdiagnosis of rare diseases is crucial and necessary to improve patient outcomes, which requires further discussion.

**Table 1:** Summary of published mural nodules of anaplastic tumors.

**Study**	**Age**	**Stage**	**n**	**Outcome (follow-up period)**
Roma and Malpica (9)	31–43 (median, 40)	Primary retroperitoneal	3	1AWD (26 months)
				2DOD (5 and 9 months)
Zhang et al. (10)	48	IA	1	NED (12 months)
Yamazaki et al. (11)	35	IA	1	NED (15 months)
Desouki et al. (12)	20	IIIC	1	AWD (-6 months)
Mhawech-Fauceglia et al. (8)	36	IA	1	DOD (15 months)
Provenza et al. (13)	15–93 (mean, 44)	IA	11	1DOC, 10NED (median, 5 years)
		IC	3	3DOD (1 month−3 years, median, 8 months)
		II	1	DOD
		III	3	1AWD, 1DOD
		IV	2	1AWD, 1DOD
		Unstage	1	DOD
Provenza et al. (13)	18–75 (median, 38)	IA	10	6NED (12–24 months), 4DOD (12–120 months)
		IC	2	1NED (30 months), 1DOD (6 months)
		II	1	1AWD (36 months), 2DOD(5–9 months)
		III	3	NED (month)
Mesban Ardakani et al. (14)	22–68 (median, 46)	IC	1	DOD (3 months)
Okumura et al. (5)	53	IIIB	1	NED (36 months)
Chaudet et al. (15)	24–81 (mean, 52)	IA	6	6NED (9–29 months)
		IC	2	2NED (37–66 months)
		II	1	NED (228 months)
		III	4	1AWD (n/a), 1DOC (post-op), 2DOD (4 and 15 months)
		IV	4	2AWD (3–7 months), 2DOD (4 and 10 months)
Current study	26–71 (median, 49)	IA	5	3 NED (6–279 months), 1DOC (121 months), 1DOD (9 months)
		IC	1	NED (11 months)
		III	3	1NED (20 months), 2DOD (3 and 8 months)
Total reported		IA	35	27NED, 2DOC, 6DOD
		IC	9	4NED, 5DOD
		II	3	1NED, 2DOD
		III	17	5NED, 1DOC, 3AWD, 8DOD
		IV	7	4AWD, 3DOD
		Total	75	

*AWD, alive with disease; DOD, dead of disease; DOC, dead of other courses; n/a, not available; NED, no evidence of disease*.

## Data Availability Statement

The original contributions presented in the study are included in the article/supplementary material, further inquiries can be directed to the corresponding authors.

## Ethics Statement

This report was approved by the Affiliated Hospital of Guangdong Medical University. Written informed consent was obtained from the patient for publication of this case report and any accompanying images.

## Author Contributions

AF and HH performed the operation. XZ, ZM, and YC were involved in the acquisition of data and preparing the figures. XZ, HH, QZ, ZM, YC, WW, and AF wrote the manuscript. QZ revised the manuscript. All authors read and approved the final manuscript.

## Conflict of Interest

The authors declare that the research was conducted in the absence of any commercial or financial relationships that could be construed as a potential conflict of interest.

## Publisher's Note

All claims expressed in this article are solely those of the authors and do not necessarily represent those of their affiliated organizations, or those of the publisher, the editors and the reviewers. Any product that may be evaluated in this article, or claim that may be made by its manufacturer, is not guaranteed or endorsed by the publisher.
